# Emotional empowerment through information research and communication to reduce fear of COVID-19 among nursing students: a randomised controlled trial

**DOI:** 10.1186/s12912-023-01370-1

**Published:** 2023-06-19

**Authors:** L. Iván Mayor-Silva, Guillermo Moreno, Pedro R. Girón-Daviña, Samir Mohamedi-Abdelkader, Marta M. Hernández-Martín, Antonio G. Moreno-Pimentel, Alfonso Meneses-Monroy

**Affiliations:** 1 Faculty of Nursing, Physiotherapy and Podiatry, Complutense University of Madrid (UCM), Pl. de Ramón y Cajal, 3, 28040 Madrid, Spain; 2 Hospital 12 de Octubre Health Research Institute (imas12), Madrid, Spain; 3 Faculty of Statistical Studies, Complutense University of Madrid (UCM), Madrid, Spain

**Keywords:** Psychological resilience, Nursing students, Self-care, COVID-19, Fear, Anxiety

## Abstract

**Background:**

Despite an existing body of literature on anxiety reduction using multi-component methods, little is known about the effect of active student participation in research and communication of scientific information on anxiety and fear reduction. The aim of this study is to evaluate the impact of quality scientific information research and the production of informative videos on the preventive aspects of COVID-19 on fear and anxiety reduction.

**Methods:**

A randomised controlled trial was conducted with 220 undergraduate nursing students in the first year of the nursing degree. The participating students were randomised into two groups. The experimental group carried out an intervention based on a database search for information on preventing COVID-19 and production of a video giving scientific reasons why prevention measures should be followed. In the control group, students created posters and videos about theoretical aspects of one module of the nursing degree. Both groups were surveyed before and after the intervention, measuring their state of resilience, preventive behaviours, level of anxiety, and fear of COVID-19.

**Results:**

The intervention group showed a greater decrease in fear levels after the intervention than those in the control group. There were no differences between the groups in terms of resilience, preventive behaviours, or anxiety. In the experimental group, there was a significant decrease in anxiety levels and fear levels after the intervention compared to the baseline levels.

**Conclusions:**

An intervention based on active participation in searches for high-quality scientific information and production of information videos on COVID-19 prevention reduced fear and anxiety caused by COVID-19 among nursing students.

**Trial registration:**

We have retrospectively registered the trial in Open Science Framework and the identification number is 10.17605/OSF.IO/6QU5S.

**Supplementary Information:**

The online version contains supplementary material available at 10.1186/s12912-023-01370-1.

## Background

During the COVID-19 pandemic, misinformation and information overload, as well as changes in social and family life, required a high level of resilience to cope with the fear and anxiety reactions triggered by the situation in the general population and particularly in nursing students [1–4], who experienced sleep disturbance, stress, severe anxiety, moderate fear and some degree of depression [5–7].

Fear is a defence and preparedness mechanism for responding to potentially threatening events; however, when it is chronic, it becomes a key trigger for a number of psychiatric disorders [8]. In the case of COVID-19, fear is directly associated with the high transmission rate and medium (rapid and invisible) and with the disease’s morbidity and mortality rates. Individuals with high levels of anxiety may not be able to cope effectively with COVID-19. However, current global management of COVID-19 has focused primarily on infection control, an effective vaccine, and cure rates with treatment [9, 10]. Notably, among nursing students during the COVID-19 pandemic, anxiety was significantly associated with gender, with females expressing more anxiety and fear than their male counterparts. Lack of personal protective equipment and fear of infection were additional factors that increased anxiety among nursing students. However, it is worth noting that resilience and the use of humour emerged as significant protective factors, as they were strongly associated with lower levels of anxiety [11, 12].

Resilience is defined as a measure of the ability to cope with stress that results from a combination of different individual characteristics. It has been shown to be linked to successful adaptation. Studies related to individuals in adverse situations reveal that while some show considerable adaptive difficulties, others are able to maintain adequate levels of performance and a degree of wellbeing in even the most difficult circumstances. Keener et al. recommend resilience training to improve quality of life and maintain clinical performance among health professionals [13]. High resilience scores were also associated with better self-care in samples of patients requiring long-term care (as in the case of diabetes mellitus) [14]. For Zager et al. resilience appears to be an important protective factor for individuals to manage stressful situations such as the COVID-19 pandemic and the lockdowns imposed for public health reasons [15]. In undergraduate nursing students, a positive relationship has been observed between resilience and various aspects of performance, such as successful completion of work placements. Resilience has also been shown to be positively associated with improved quality of life and general wellbeing in these students [16, 17].

Anxiety is “*the feeling of fear that occurs when faced with threatening or stressful situations. It is a normal response when confronted with danger, but, if it is overwhelming or the feeling persists, it could be regarded as an anxiety disorder*” [18]. In addition, other factors such as anxiety may lead to greater use of self-protective measures by individuals in the face of COVID-19 and those who trivialise its consequences. It was found that higher levels of anxiety symptoms since the onset of the outbreak were a strong and significant factor influencing the adoption of preventive measures [19]. Furthermore, during the early months of the pandemic in Wuhan, China, it was reported that younger people had higher levels of anxiety than older people. In addition, the majority of respondents were diligent in following the preventive measures prescribed by the health authorities [20]. In a comprehensive survey conducted in Japan, individuals who took the most stringent preventive measures were characterised by a high level of vigilance towards the risk of COVID-19 and a high level of anxiety about the possibility of infection [21].

Although nursing students have access to reliable sources of scientific information, some select social media as their primary source of information on COVID-19 [22]. This may be related to the fact that nursing students believe that the use of technology, especially mobile technology, can improve their learning experience [23]. However, during the COVID-19 lockdown in Spain, students’ knowledge about COVID-19 transmission was inaccurate or inadequate [24]. This could indicate that the quality of the information they receive through social media is insufficient.

Current recommendations encourage university lecturers to help students search for information through institutional databases and provide training on the latest developments in information and communication technology [25]. Making educational videos using narrated digital stories has proven to be a good teaching and learning strategy to reinforce the main thematic units of nursing studies [26]. In line with these recommendations, our aim is to implement an intervention that focuses on ensuring access to accurate and reliable information, using informative videos that address preventive measures. The ultimate goal is to debunk the common myths and misconceptions surrounding the disease and to reduce the anxiety and persistent fears associated with it. In this way, nursing students can gain greater self-confidence and knowledge and carry out interventions to reduce fear and anxiety among the population.

## Methods

### Study design. Population and participants

A randomised controlled trial study with a two-arm, parallel design (1:1) was conducted in November 2020 (all participants were recruited on 2nd of November and followed until 30th of November). The study design, conduct, and analysis followed the recommendations set out in the CONSORT statement [27].

The inclusion criteria were: any first-year undergraduate nursing student at the Faculty of Nursing, Physiotherapy and Podiatry, Complutense University of Madrid (UCM) who accepted the conditions of the study by giving their informed consent. All students were enrolled in the subject of psychology in which the study was carried out. Students who did not accept the conditions of the study, did not sign the informed consent form, left the session before its completion or submitted a null questionnaire, and those who alleged personal health reasons for not participating were excluded. Finally, 220 students were selected and randomly assigned to one of the two homogeneous groups in terms of the number of participants (ratio 1:1). For the randomisation, a list of random numbers was generated by one of the researchers using the statistical and epidemiological programme Epidat 4.2. At the beginning of the intervention, both groups had to sign a written agreement not to disclose the content and objectives of the research. This measure was taken to control and prevent any possible cross-contamination effect.

**Fig. 1:**
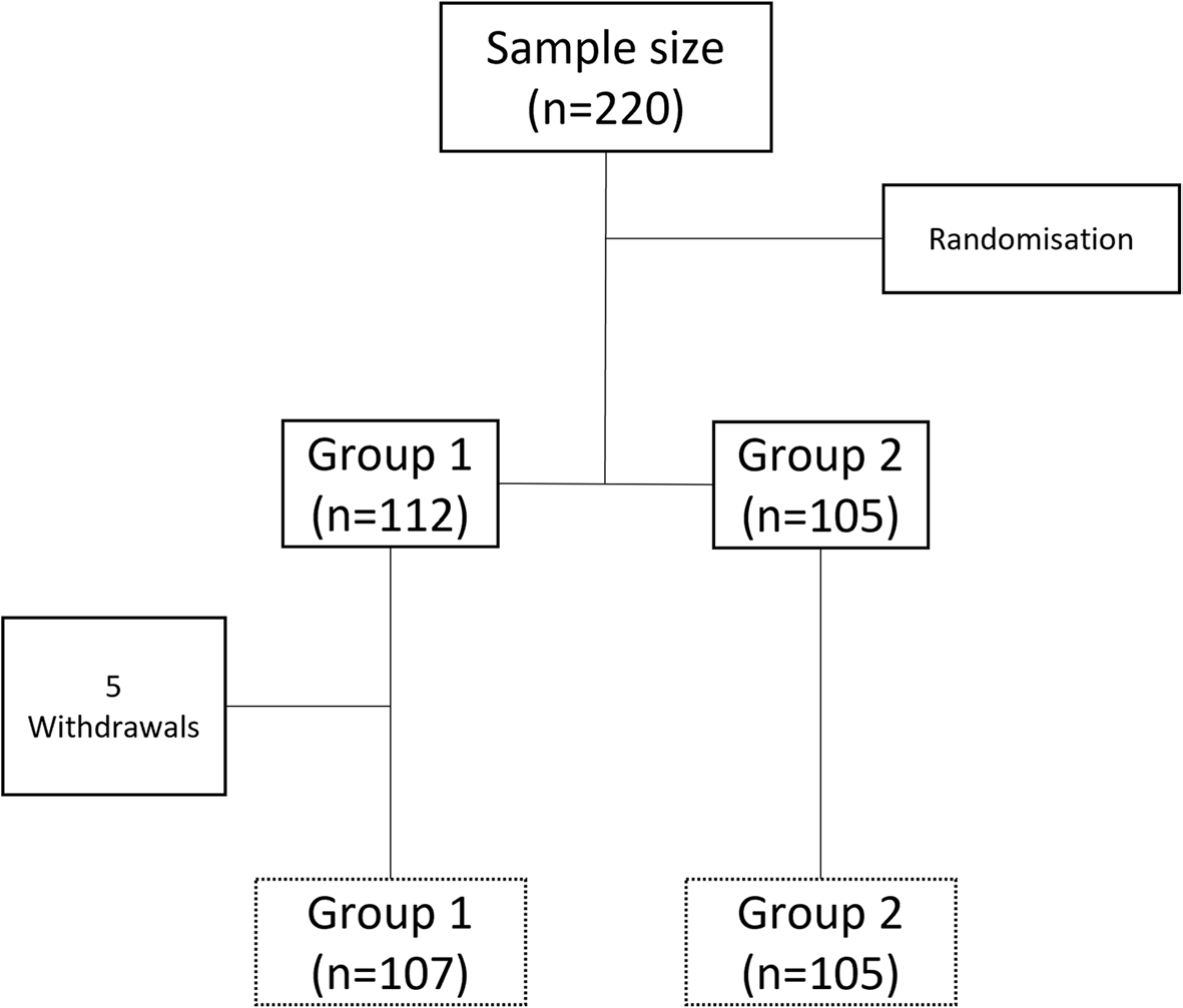
Flowchart of study participants

One hundred seven students in the intervention group (Group 1) and 105 students in the control group (Group 2) completed the study (Fig. 1).

### Scales of measurement and variables

All participants were given a series of validated, self-report questionnaires one week before and one week after the intervention. The post-intervention assessment was carried out one week after the end of the intervention to ensure that students had sufficient experience to be able to apply what they had learned on a day-to-day basis in the context of a pandemic. By giving the students enough time, we gave them the opportunity to deeply process what they had learned, which could lead to a change in their beliefs and attitudes, and this change could have an impact on their emotions. Sociodemographic data were collected from all participants, including control variables (sex, age, education, employment status, and marital status).

The questionnaires assessed a range of psychological factors. The validated, self-report questionnaires used for this purpose included:

1. The Connor-Davidson Resilience Scale (CDRS) (Spanish adaptation by Crespo et al., 2014) [28] is a Likert scale ranging from 0 (not at all) to 4 (almost always), consisting of 25 items grouped into 5 dimensions: persistence-tenacity-self-efficacy, control under pressure, adaptability and support networks, control and purpose, and spirituality. The sum of all of the items constitutes the total Resilience value, whose cut-off points are < = 70 (low), 70–87 (intermediate), and > = 88 (high). The internal consistency of the Spanish version was optimal, with a Cronbach’s alpha coefficient = 0.86 [29].
2. The Coronavirus Anxiety Scale (CAS) [30] contains 5 items scored on a scale of 0 to 4 according to symptom frequency, with 0 being ‘never’ and 4 being ‘almost every day’. These items assess different manifestations of anxiety in relation to coronavirus, taking a variety of areas into consideration: cognitive (repetitive thinking, worry), behavioural (poor functioning, compulsive or avoidant behaviour), emotional (fears and distress) and psychological (sleep-related problems). Internal consistency was good for the Spanish version: ω = 0.89; ordinal α = 0.89 [31].
3. The Coronavirus Fear Scale (CFS) is a four-factor questionnaire on coronavirus-related fears: (F1) Fear of contagion, illness, and death; (F2) Fear of lack of basic consumer goods; (F3) Fears of social isolation; and (F4) Fears related to work and income, designed to assess the fears and concerns experienced by the subject during the COVID-19 pandemic. It contains 18 items relating to fears and concerns about different psychosocial aspects of coronavirus that are rated using Likert response options: Not at all or hardly at all = 1, A little = 2, Quite a lot = 3, A lot = 4; and Very much or extremely = 5. Internal consistency was α = 0.89 [32].
4. Use of Preventive Behaviours (UPB) is a questionnaire with 7 behaviours to avoid coronavirus infection described by health authorities. Each statement is answered true or false [32].

Another factor assessed was contact with the virus in family members with the following response options: No close contact with a person with COVID-19; With mild symptoms; With severe symptoms or Death from COVID-19.

### Procedure

The independent variable is a pedagogical intervention based on an active methodology that offers benefits at an attitudinal and learning level [33]. Project-based learning is a methodology that allows students to acquire knowledge and key competences in the 21st century by working on projects that reflect real-life problems [34]. The method used in this active learning study was the creation of informative videos.

In both groups (intervention and control), the process was structured into the following phases:

- **Phase 1**: Scientific information research. Training of students in search strategies for scientific information in scientific databases (i.e., Pubmed, Web of Science). We provide students with video and PDF content on the following topics: access to scientific information online, advantages and disadvantages, search engines, specialised nursing search engines, multidisciplinary databases and evaluating journal quality.
- **Phase 2**: Communication through informative videos. Students were taught to convey relevant information in terms accessible to the general population through digital media. To develop their skills, the students used a technique for presenting doctoral theses in 3 min, following the instructions in the video *3 TRICKS TO WIN (3MT)* [35].
- **Phase 3**: Training on creating a scientific poster. Students were taught to complete a template in scientific poster format, with a particular emphasis on the content, use of language, figures and references. The aim of this task was to develop the student’s ability to synthesise information effectively [36].
- **Phase 4**: Content creation phase. This phase involved the practical application of the skills acquired in the previous phases, namely the search for scientific information, the synthesis of this information into a scientific poster and its subsequent dissemination through videos. Students were asked to gather information using a specific media, in this case a scientific poster, and communicate it to others using video. Students in the intervention group created content aimed at informing the population on relevant aspects concerning COVID-19, giving scientific reasons why prevention measures should be followed. The students in the control group created content related to topics from the Psychology subject in the first year of the Bachelor’s Degree in Nursing. The selection of these topics for both the experimental and control groups was a collaborative effort involving 6 faculty members (including psychologists and nurses), a group of 5 students (who did not take part in the study), and 2 librarians in a discussion group (Supplemental Table 1). The topics chosen for the control group were those typically covered in the psychology subject in which the students were enrolled, including behaviour modification techniques and emotional regulation skills. These topics were included specifically to provide students with tools to improve their ability to regulate their levels of anxiety and fear within the context of this educational intervention. The topics were randomly assigned to the groups. The students had one month to make their videos and upload them to the university platform for grading. Once the research project was completed, all students (experimental and control group) had access to the video content.
- **Phase 5**: Evaluation of the students’ work. Finally, the videos were evaluated by the students themselves according to a Likert scale (from 0 to 5) agreed upon by the professors of the subject and homogenised by the teachers of the subject and the participating researchers. The aim of this phase was the self-observation of the students in order to improve their performance and consolidate the content acquired.

For the purposes of this intervention, and to avoid potential disruptions, it was delivered in person to both groups via synchronous videoconferencing. The content was made accessible to the students at all times, with a particular focus on searching for scientific information and developing materials that were common to both groups.

### Ethical and legal aspects

The authors assert that all procedures contributing to this work comply with the ethical standards of the relevant national and institutional committees on human experimentation and with the Helsinki Declaration of 1975, as revised in 2008. All procedures involving human subjects/patients were approved by the Ethics and Research Committee of the Faculty of Nursing, Physiotherapy and Podiatry (approval number: FEFP 20/21, on 4 November 2020). All students included in the study were informed, verbally and in writing, of the study objectives and conditions. Written informed consent was obtained from all participants. It was explained that participation was completely voluntary and anonymous, that they were free to withdraw from the study whenever they wished, and that their participation did not entail any benefit or harm to the student. The confidentiality and privacy of the information was respected in compliance with national regulations on personal data protection. The data were securely stored in databases and access was limited to research personnel only. The data were analysed solely for the purposes of the study. This project was retrospectively registered in Open Science Framework (OSF) according to the ICMJE guidelines at 09/08/2022, identifier 10.17605/OSF.IO/6QU5S.

### Data analysis

Descriptive analysis was performed using proportions and number of events for qualitative variables and mean and standard deviations for quantitative variables. To describe the baseline characteristics of all the variables, chi-squared tests were used, with the exception of age, where an Analysis of Variance (ANOVA) was employed (independent samples *t*-test, Mstudent). ANOVA was also used to identify potential relationships between the different measurement scales before and after the intervention and to analyse possible differences in the groups to be compared with each scale before and after the intervention. If differences were found between groups, the Bonferroni multiple comparison test was used.

To study the effect of the intervention on the different scales, an Analysis of Covariance (ANCOVA) was carried out. Results were expressed as means, *p*-values, and 95% confidence intervals.

## Results

### Description of the sample

Of the total students who completed the study, 84.3% were female (three out of four were between 18 and 19 years old), approximately 8 out of 10 were single, and 22.8% of the students were studying and working at the same time. One in four participants had had close contact with an individual with severe symptoms of COVID-19 since the start of the pandemic, 40.8% of participants had come into contact with an individual with mild COVID-19 symptoms, and 33.7% had had no contact at all with anyone with COVID-19 (Table 1). There were no statistical differences between groups regarding baseline levels of anxiety, fear, resilience, and preventive behaviours.

**Table 1:** Sociodemographic characteristics of the sample

		Total	Experimental Group	Control Group	*p*-value
		*n* = 212	*n* = 107	*n* = 105	
**Variable**		**Mean or n (SD or %)**	**Mean or n (SD or %)**	**Mean or n (SD or %)**	
**Sex**	Female	166 (84.7%)	84(84.0%)	82 (85.4%)	0.745
	Male	30 (15.3%)	16(16.0%)	14 (14.6%)	
	No response	16	7	9	
**Age in years**		20.1 (5.7)	20.4 (6.1)	19.8 (5.2)	0.494
**Marital status**	Single	167 (86.1%)	84 (84.8%)	83 (87.4%)	0.586
	No Single	27 (13.9%)	15 (15.2%)	12 (12.6%)	
	No response	18	8	10	
**Employment status**	Unemployed	168 (86.1%)	87 (87.0%)	81 (86.2%)	0.921
	In the healthcare sector	13 (6.1%)	6 (6.0%)	7 (7.4%)	
	In another sector	13 (6.1%)	7 (7.0%)	6 (6.4%)	
	No response	18	7	11	
**Close contact with a person with COVID-19**	No contact	63 (33.5%)	29 (30.2%)	34 (37.0%)	0.395
	Mild symptoms	78 (41.5%)	44 (45.8%)	34 (37.0%)	
	Severe symptoms	47 (25.0%)	23 (24.0%)	24 (26.1%)	
	No response	24	11	13	

### Behavioural scales after intervention

**Fig. 2:**
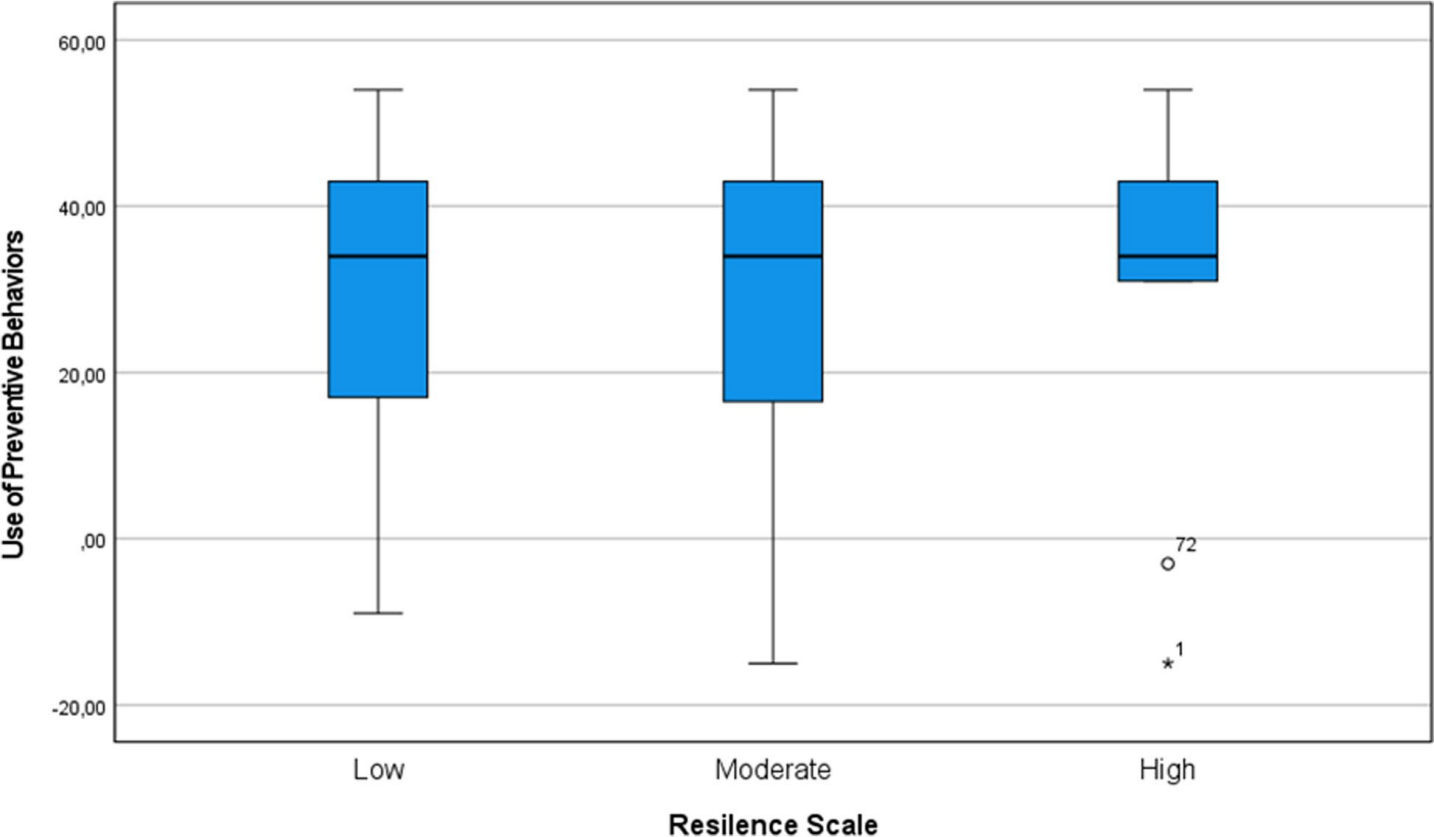
Performance of the use of preventive behaviours by resilience status after the intervention

In the analysis of the relationship between preventive behaviours, anxiety levels, fear of COVID-19, and resilience, no associations were found. After the intervention, regardless of the level of resilience, the mean value on the Use of Preventive Behaviours scale was similar (Fig. 2).

**Fig. 3:**
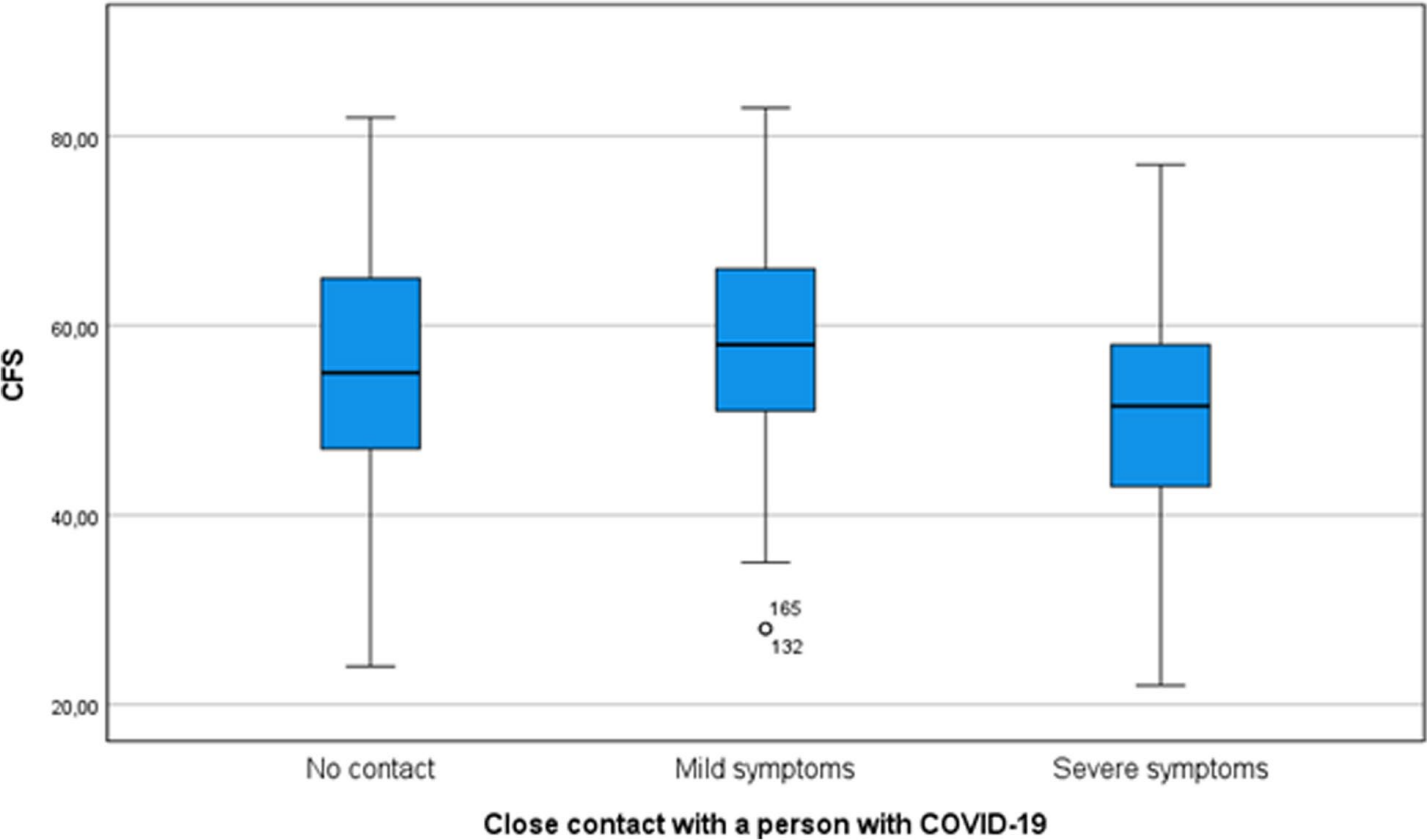
Coronavirus fear scale by degree of contact with an individual with COVID-19 after the intervention. CFS: Coronavirus Fear Scale

Having been in close contact with a case of COVID-19 since the beginning of the pandemic is associated with the CFS scale only (*p*-value < 0.01). No differences were identified between students who had not come into close contact with anyone with COVID-19 and those who had come into contact with individuals with mild or severe symptoms (Fig. 3).

### Effect of the intervention within and between groups

Given the variable difference of each scale pre- and post-intervention (dScale = Scale-Scaleprepost), we analysed how the scales performed within each group and between groups.

Post-intervention, in the experimental group (Group 1), there was a slightly significant decrease in anxiety levels and this decrease is more noticeable when we consider the assessment of fear levels. In the control group (Group 2), no changes were observed in any of the scales used (Table 2).

**Table 2:** Performance of scales after-before in each group

	95% CI (after-before) *p*-value			
Scale	**Group 1**	*p*-value	**Group 2**	*p*-value
**CD-RISC**	(-0.79,2.58)	0.30	(-1.24,1.89)	0.70
**CAS**	(-0.93,-0.01)	0.04	(-1.00,0.06)	0.08
**CFS**	(-4.91,-1.85)	< 0.01	(-2.19,0.98)	0.50
**UPB**	(-3.10,2.55)	0.80	(-0.61,5.14)	0.10

**Fig. 4:**
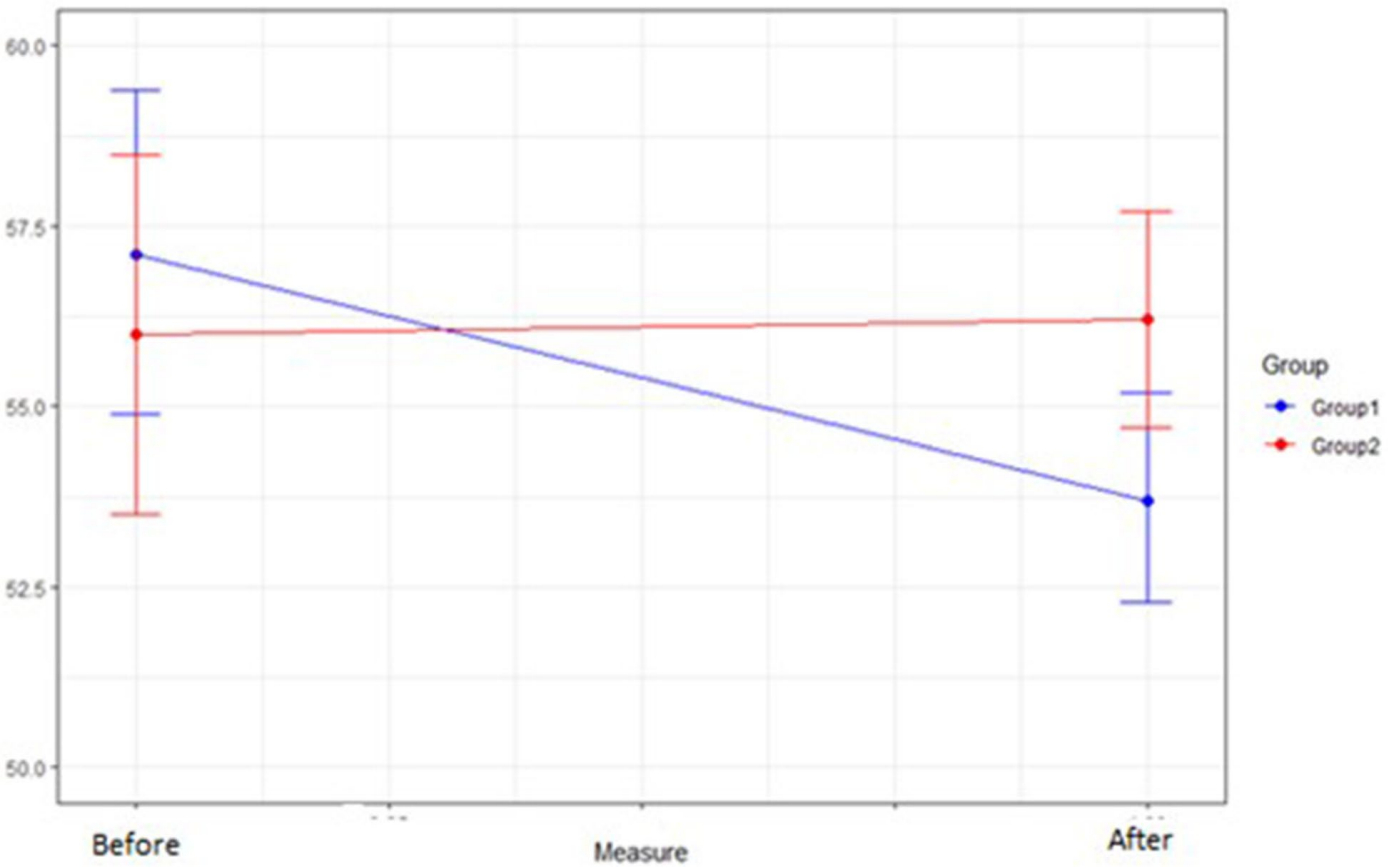
Effect of the intervention on the CFS scale. CFS: Coronavirus Fear Scale

Regarding the ANCOVA on the CFS scale, the students in the intervention group had a different score on the CFS after the intervention (*p* = 0.025) to those in the control group. Considering the confidence intervals of the mean fear score after the intervention, it can be stated with 95% confidence that the mean level of fear in the intervention group (53.7 points 95%CIG1 52.3–55.2) is lower than that of the control group (56.2 points 95%CIG2 54.7–57.7) (Fig. 4).

Comparing the behaviour and effect of the different dimensions (F1, F2, F3, F4) of the CFS scale after the intervention, only differences in dimensions F2 and F4 were observed between the two groups after the intervention.

**Fig. 5:**
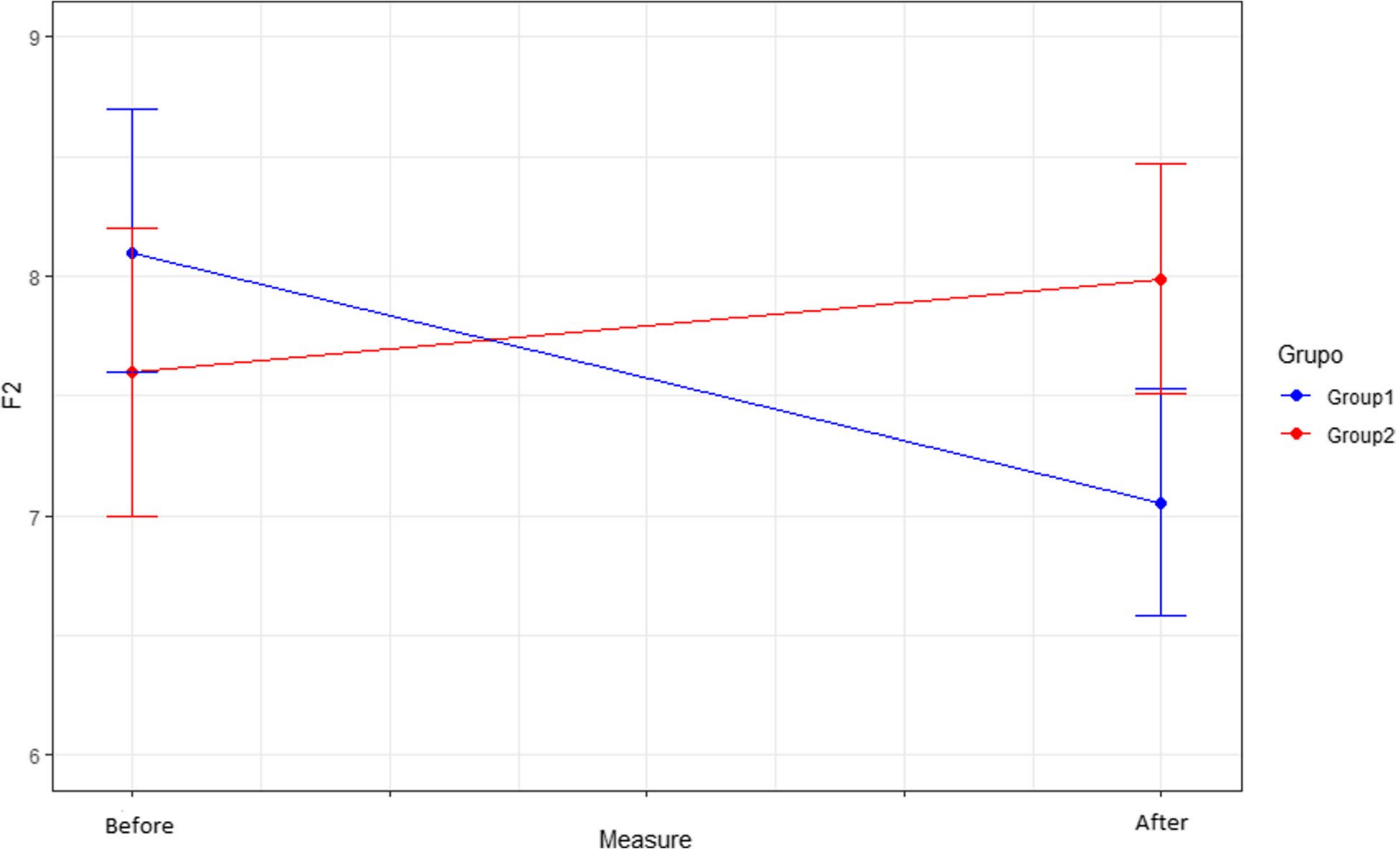
Effect of the intervention on F2. F2: Fear of lack of basic consumer goods

**Fig. 6:**
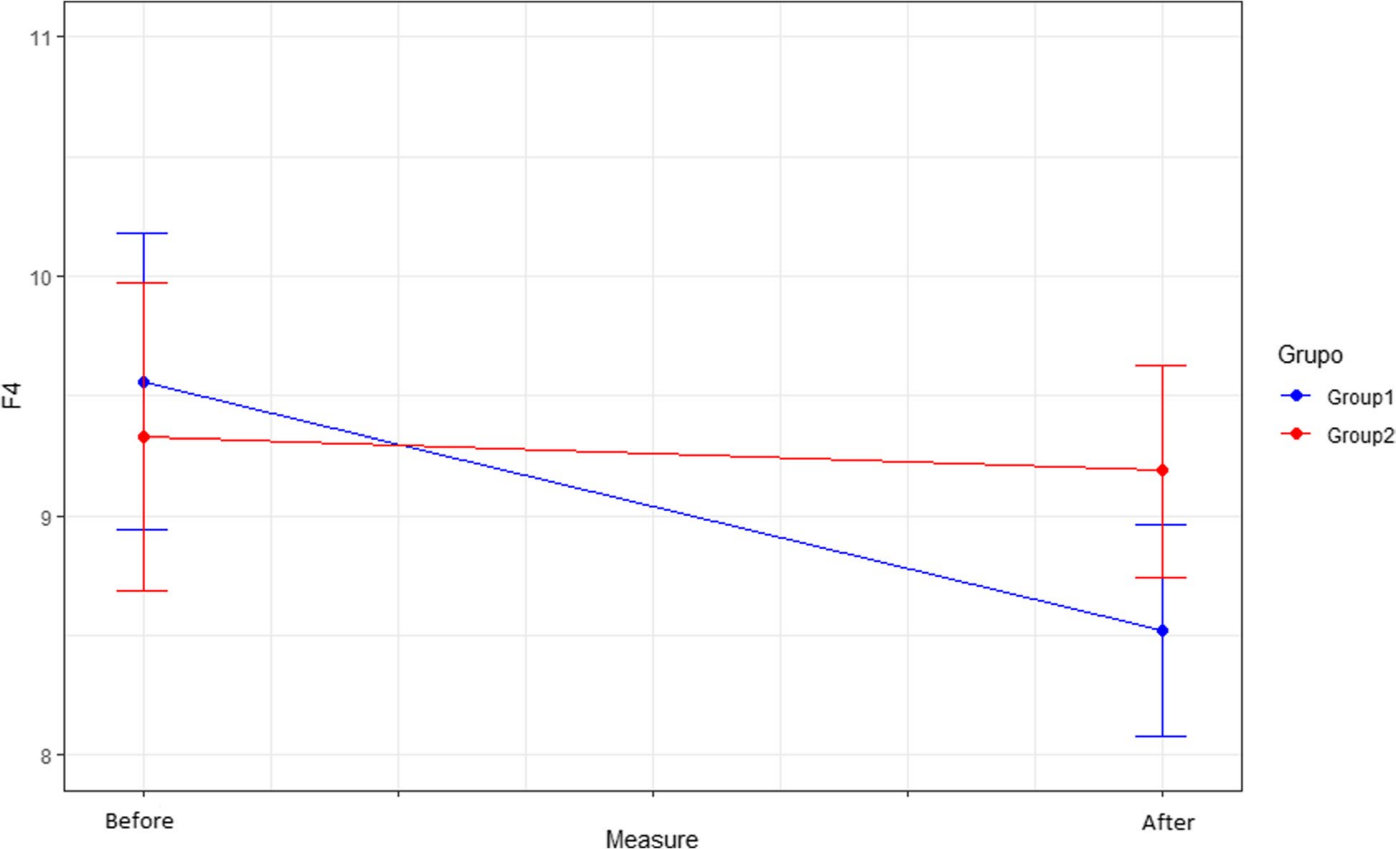
Effect of the intervention on F4. F4: Fears related to work and income

In F2 (Fear of lack of basic consumer goods), the mean score in the intervention group (7.0 points) is lower than in the control group (7.9 points) after the intervention (*p* = 0.007). In dimension F4 (Fears related to work and income), the mean score in the intervention group is significantly lower than in the control group after the intervention (*p* = 0.037). The mean score for F4 after the intervention in the intervention group is 8.5 points compared to 9.56 before the intervention, while in the control group the mean score does not change (9.33 points) (Figs. 5 and 6).

## Discussion

Our results show that students who had come into close contact with an individual with severe COVID-19 symptoms obtained a higher mean score on the CFS than those who had come into contact with individuals with mild COVID-19 symptoms or had had no contact at all. However, after the intervention, the students who had come into close contact with an individual with severe COVID-19 symptoms obtained a lower mean score. This is consistent with Kabasakal et al., who conclude that there is a significant correlation between having family members with COVID-19 or losing a relative to COVID-19 and fear of COVID-19 among service sector employees [37]. However, in our study, this correlation was not significant among healthcare workers, perhaps because they had more information, which may explain why fear decreased post-intervention.

After the intervention, there was a decrease in mean levels of anxiety and fear. These results are explained by Medina et al., who observe that the presence of high stress levels and low knowledge levels predict fear of COVID-19. They conclude that there is a need for intervention on knowledge, stress, and fear of COVID-19 in the study population, as was the case in the present study [38].

Other studies have found that nursing students have moderate levels of fear and mild levels of anxiety during their clinical placements [6]. However, to our knowledge, this is the first study that has used an educational method to decrease levels of emotional distress (fear) through information seeking and video production. Previous studies have observed that fear levels among nursing students in their final year were higher compared to those in their first year [39]. This suggests that contact with patients may not be a variable that effectively reduces fear, which aligns with the findings of our study (as depicted in Fig. 4). It is important to acknowledge that the authors of our study did not directly intervene with the students. Most intervention studies involving online tools in nursing students have employed psychological intervention techniques [40]. Therefore, future studies should explore the impact of emotional learning and the acquisition of knowledge through traditional classroom settings to gain a comprehensive understanding of their effects.

It may be interesting to use this methodology with nursing students to reduce fears about some key aspects of their future work performance. This will not only consolidate their knowledge but will also give the students a greater sense of certainty and empowerment to banish their fears and those of the people around them.

It should be noted that although the students in the control group had to work on psychological content related to the basics of behaviour control therapy and emotional regulation, no transfer of knowledge about their behaviour was observed. It seems that working on content directly related to the illness is more effective in improving emotional regulation than teaching general knowledge about behaviour regulation. This finding contradicts the existing literature, which suggests that techniques such as mindfulness have a positive effect on nursing students by reducing their anxiety levels. However, it is important to note that the focus of these interventions is not on knowledge transfer as in our intervention. The strategies used in the interventions found in the literature have limited efficacy, possibly due to their small effect size and the relatively small sample size of students [41, 42].

Bakioğlu et al. and Begum et al. [43, 44] explain that fear is a strong emotion affecting individuals’ physical responses, cognitive abilities, and mood, generating greater worry and aggravating the severity of the situation experienced by the individual. The impact of fear on mental health among nursing students and professionals can be severe, as they experience constant psychological distress caused by their work on the frontline, interact with staff who are in contact with the virus, and worry about their uncertain professional futures.

It is also important to highlight that video production was used as a means for acquiring knowledge in this study and this appears to generate greater confidence among students. According to Rodríguez-Almagro et al., nursing students acknowledge that this training activity helps them to feel better prepared and to better understand the module in question based on the real-life nature of their work [26]. Our study corroborates Hossain et al., who conclude that higher levels of knowledge reduce levels of fear of COVID-19, which could support the hypothesis that lack of knowledge about a specific area encourages ingrained misconceptions and misbeliefs [45]. According to McEachan et al., knowledge of the disease is a prerequisite for establishing prevention behaviours, forming positive attitudes, and promoting positive behaviours and thoughts. People’s attitudes towards the disease also affect the effectiveness of their coping strategies and behaviours to some extent [46]. However, we cannot draw clear conclusions about which component is responsible for the effect of the intervention. The most likely explanation for this effect is the processing of the content, which could change students’ attitudes towards the disease and therefore their behaviour.

Although there are examples of interventions that aim to change attitudes through knowledge acquisition [47], there are other interventions that demonstrate attitude change through active learning [48]. However, we found evidence in the literature that access to information about COVID-19 among students does not translate into a high number of effective behaviours and attitudes towards COVID-19, particularly when students are not related to health science careers [49]. This may support the hypothesis that access to information is not enough. However, the design of our study does not allow us to fully explain whether the effect observed in the experimental group could be achieved with another type of intervention focusing solely on classical lectures and knowledge acquisition.

In future studies, it would be interesting to test whether other knowledge acquisition tools (used in the academic context), such as infographics [36], can reduce the levels of negative emotions associated with certain diseases and improve preventive behaviours. It would also be of interest to verify whether our results on preventive behaviours, resilience, fear, and anxiety are similar among other health science students, and whether interventions based on the use of audio-visual media are effective in reducing fear in these students. Furthermore, it would be intriguing to clarify the specific components of this intervention that have the most significant impact on emotional change. In addition, exploring the potential influence of other interventions that focus on the acquisition of knowledge about the disease, such as the use of written materials for student study, could contribute to our understanding of how attitudes and emotions are influenced.

### Limitations of the study

The questionnaires and scales used were self-reports, which may have introduced social desirability and subjectivity bias, resulting in potentially distorted responses. Theoretical limitations include the fact that there are few studies relating to the variables evaluated in this research, so the results obtained cannot be extrapolated to the general population, although they are valid for the university analysed in this study. In addition, most interventions on fear and stress tend to last several hours and use multi-component programmes and mindfulness, while ours consisted solely of searching and disseminating information. On the other hand, we failed to identify why this intention did not improve other fears. In this regard, instead of randomising the topics dictated by the teachers, the topics relating to the pandemic that were of greatest concern to the students should perhaps have been used instead.

Based on the aforementioned results, it can be concluded that although levels of resilience and use of preventive behaviours do not change throughout the study, the level of anxiety decreases after the intervention in the intervention group, but there is no evidence that their mean anxiety level differs from that of the control group. However, the level of fear of coronavirus in the intervention group decreases post-intervention and its mean value is lower than in the control group post-intervention. This results in lower scores on Fear of lack of basic consumer goods (F2) and Fears related to work and income (F4). Programmes aied at students to banish fears about certain aspects of clinical practice can improve their sense of empowerment and allow them to acquire more solid, evidence-based knowledge and become good health promoters in their immediate environments.

### Implications for practical uses

Our results suggest that audio-visual media should be used to reduce fears in modules aiming to equip students with health prevention skills (such as Public Health). Thanks to these audio-visual methods and the change of emotions they generate, students could act as role models for the people around them. This could also benefit students during their clinical placements by reducing their biological risk of contagion.

## Conclusions

Involving active participation in searching for scientific information and creating information videos on COVID-19 prevention, effectively reduced fear and anxiety among nursing students. This comprehensive approach empowered students with accurate knowledge, improved their communication skills, and boosted their confidence in facing the pandemic. By engaging in knowledge dissemination, students took on the responsibility of sharing reliable information. This intervention highlights the importance of active engagement and knowledge sharing in reducing negative emotions, fostering resilience and critical thinking. Further research is needed to identify key intervention components and explore long-term effects on emotional well-being and attitudes towards future public health crises.

## Supplementary Information

**Additional file 1.**

## Data Availability

The datasets generated and/or analysed during the current study are not publicly available due to data protection policy but are available from the corresponding author on reasonable request.

## Abbreviations

CDRS: Connor-Davidson Resilience Scale
CAS: Coronavirus Anxiety Scale
CFS: The Coronavirus Fear Scale
UPB: Use of Preventive Behaviours
