# Supplementary material for: Emotional empowerment through information research and communication to reduce fear of COVID-19 among nursing students: a randomised controlled trial
Source: BMC Nurs. 2023 Jun 19;22:209. doi: 10.1186/s12912-023-01370-1 (PMC10278329; doi:10.1186/s12912-023-01370-1)
Supplement: Supplementary file 1 — Additional file 1. [file 12912_2023_1370_MOESM1_ESM.docx]

**SUPPLEMENTAL MATERIAL**

**Supplemental Table 1. List of topics (intervention and control group)**

| **Intervention Group Topics** |
| --- |
| 1. Mechanisms of transmission of COVID-19 |
| 1. Protection measures against COVID-19 |
| 1. More effective masks against COVID-19 |
| 1. What is the current status of vaccines against COVID 19? |
| 1. What are the COVID-19 Treatments that have been shown to be most effective? |
| 1. What are the populations at risk or most vulnerable to complications from COVID 19? |
| 1. What has been the evolution of the COVID 19 pandemic in Spain and in the world? |
| 1. Protection measures in educational centers |
| 1. Pathophysiology of infection |
| 1. Most common symptoms by age group |
| 1. Atypical manifestations of COVID 19 |
| 1. Long-term evolution of COVID 19 |
| 1. Reinfection of COVID 19 |
| 1. Coinfection with COVID 19 |
| 1. Neurological disorders due to COVID 19 |
| 1. Relationship between the immunity produced by the flu versus COVID 19 |
| **Control Group Topics** |
| 1. What are short-term memory disorders? |
| 1. Long-term memory disorders |
| 1. Thought disorders |
| 1. Language disorders |
| 1. Sensoperception |
| 1. Classical conditioning in addictions |
| 1. Operant conditioning in changing habits |
| 1. Behavioral Medicine |
| 1. Social psychology and attitudes towards illness |
| 1. Applications of hypnosis for health |
| 1. Drugs and altered states of consciousness |
| 1. Optical Illusions |
| 1. Mindfulness applications for stress control |
| 1. Mindfulness applications for depression |
| 1. Evaluation of suicidal behavior |
| 1. Emotional disorders |
